# A Brain Region-Based Deep Medullary Veins Visual Score on Susceptibility Weighted Imaging

**DOI:** 10.3389/fnagi.2017.00269

**Published:** 2017-08-08

**Authors:** Ruiting Zhang, Ying Zhou, Shenqiang Yan, Genlong Zhong, Chang Liu, Yerfan Jiaerken, Ruirui Song, Xinfeng Yu, Minming Zhang, Min Lou

**Affiliations:** ^1^Department of Neurology, The Second Affiliated Hospital of Zhejiang University, School of Medicine Hangzhou, China; ^2^Department of Radiology, The Second Affiliated Hospital of Zhejiang University, School of Medicine Hangzhou, China

**Keywords:** deep medullary veins, white matter hyperintensities, susceptibility-weighted images, venous drainage regions, venous ischemia

## Abstract

Cerebral venous collagenosis played a role in the pathogenesis of white matter hyperintensities (WMHs) through venous ischemia. Since pathological changes of veins from intramural stenosis to luminal occlusion is a dynamic process, we aimed to create a deep medullary veins (DMVs) visual grade on susceptibility-weighted images (SWI) and explore the relationship of DMVs and WMHs based on venous drainage regions. We reviewed clinical, laboratory and imaging data from 268 consecutive WMHs patients and 20 controls. SWI images were used to observe characteristics of DMVs and a brain region-based DMVs visual score was given by two experienced neuroradiologists. Fluid attenuated inversion recovery (FLAIR) images were used to calculate WMHs volume. Logistic-regression analysis and partial Pearson’s correlation analysis were used to examine the association between the DMVs score and WMHs volume. We found that the DMVs score was significantly higher in WMHs patients than in controls (*p* < 0.001). Increased DMVs score was independently associated with higher WMHs volume after adjusting for total cholesterol level and number of lacunes (*p* < 0.001). Particularly, DMVs scores were correlated with regional PVHs volumes in the same brain region most. The newly proposed DMVs grading method allows the clinician to monitor the course of DMVs disruption. Our findings of cerebral venous insufficiency in WMHs patients may help to elucidate the pathogenic mechanisms and progression of WMHs.

## Introduction

White matter hyperintensities (WMHs), also known as leukoaraiosis, are commonly seen as confluent or patchy hyperintense areas on T2 weighted or fluid attenuated inversion recovery (FLAIR) scans in the elderly (Wardlaw et al., 2013). The presence of WMHs is associated with cognitive impairment and mood disorders, increases the risk of stroke, dementia and mortality (Kim et al., 2008; Debette and Markus, 2010). However, the underlying pathogenic mechanisms of WMHs is so far unclear.

Although previous studies have demonstrated that the development of WMHs was related to the damaged blood-brain barrier, disruption of capillary permeability and diminished regional cerebral blood flow (O’Sullivan et al., 2002; Brickman et al., 2009; Uh et al., 2010). It was also shown that cerebral venous collagenosis may cause venous ischemia and lead to non-necrotic hyperintensities on MRI by increasing vascular resistance and compromising interstitial fluid circulation, with consequent vessel leakage (Moody et al., 1995; Black et al., 2009). Recently, Yan et al. (2014) demonstrated increased number of voxels of deep medullary veins (DMVs) on susceptibility-weighted imaging (SWI) was independently associated with WMHs volume. However, conversely, Guio et al. (De Guio et al., 2014) observed a significant reduction in the number of visible veins within WMHs in patients with cerebral autosomal dominant arteriopathy with subcortical infarcts and leukoencephalopathy (CADASIL).

Actually, pathological changes of veins from intramural stenosis to luminal occlusion is a dynamic process (Black et al., 2009), which may result in different voxel and visibility of veins on images during different stage. The discrepant conclusions may be contributed to the difficulty in repeatability of voxel analysis. Moreover, the venous compartment is responsible for 70 to 80% of the circulatory volume inside the cranial cavity and the classification of superficial and deep cerebral venous system depends on the anatomy of drainage regions across the brain (Lee et al., 1996). Thus, it is necessary to investigate the regional association of DMVs and WMHs when considering their possible relationships. Taken them together, we deem it worthy to create a DMVs visual score on SWI and explore the exact relationship of DMVs and WMHs based on venous drainage regions.

## Materials and Methods

### Study Subjects

This was an investigator-initiated prospective single-center study. We reviewed the demographic, clinical, laboratory and radiological data of all patients who received brain MRI scan but had no diagnostic intracerebral lesions such as trauma, infection, acute stroke, and space-occupying lesions in our department from January 2010 to April 2016.

Inclusion criteria were: (1) WMHs visible on FLAIR images; (2) age above 30; (3) had written informed consent. Exclusion criteria were: (1) patients with secondary causes of white matter lesions, such as demyelinating, metabolic, immunological, toxic, infectious, and other causes; (2) patients with abnormal brain MRI findings such as head trauma, hemorrhage, infarction (except lacunes) and other space-occupying lesions; (3) patients with definitive peripheral neuropathy, spinal cord disease; (4) evidence of calcification on CT scans or encephalomalacia in the deep gray matter structures since it may influence the observation of DMVs.

Twenty normal control subjects with matched age and gender were recruited from elderly volunteers for the visibility of DMVs after giving written informed consent, who were volunteers of our ongoing functional MRI projects. Their clinical data (for common vascular risk factors such as hypertension, diabetes mellitus and hyperlipidemia), laboratory examinations, and radiological examinations (both MRI and CT) were normal.

### Ethics Statement

All subjects had given written informed consent prior to the study, and the protocols had been approved by the local ethics committee. All clinical investigation has been conducted according to the principles expressed in the Declaration of Helsinki.

### MRI Protocol

All subjects underwent multi-model MRI by a 3.0 T MR (MR750, GE Healthcare, United States) scanner using an 8-channel brain phased array coil, including T1, T2, T2 FLAIR, and SWI sequence. In order to minimize head motion, foam pads were inserted into the space between the subject’s head and the MRI head coil. Axial T2 FLAIR sequence was used to measure the WMHs volume with the following parameters: repetition time = 8400 ms, echo time = 150 ms, FOV = 24 cm × 24 cm, matrix size = 256 × 256, inversion time = 2100 ms, slice thickness = 4.0 mm with no gap (continuous) between slices, and in-plane spatial resolution of 0.4688 mm/pixel × 0.4688 mm/pixel. The whole brain was imaged. The SWI sequence was in an axial orientation parallel to the anterior commissure to posterior commissure line and covered the whole lateral ventricles, using a three-dimension multi-echo gradient-echo sequence with 11 equally spaced echoes: echo time = 4.5 ms [first echo], inter-echo spacing = 4.5 ms, repetition time = 34 ms, FOV = 24 cm × 24 cm, matrix size = 416 × 384, flip angle = 20°, slice thickness = 2.0 mm with no gap between slices, and in-plane spatial resolution of 0.4688 mm/pixel × 0.4688 mm/pixel. Flow compensation was applied. Magnitude and phase images were acquired.

### Measurement of DMVs

The raw data were transferred to a separate workstation (ADW4.4, GE), and a custom built programwas used to reconstuct the magnitude and phase images.

**FIGURE 1:**
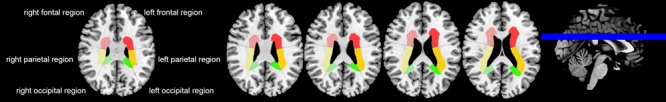
Brain regional division. Three regions were segmented including frontal region, parietal region and occipital region according to medullary venous anatomy. The colored area were PVHs of the different brain regions.

We assessed DMVs on five consecutive periventricular slices (10 mm thick) of SWI phase images from the level of the ventricles immediately above the basal ganglia to the level of the ventricles immediately disappeared for each patient, considering that these slices cover most of the DMVs. According to medullary venous anatomy (Lee et al., 1996), six regions including frontal region, parietal region and occipital region (bilateral, respectively) were separated on the above five slices and the characteristics of the DMVs were then evaluated in each region, respectively (**Figure 1**).

**FIGURE 2:**
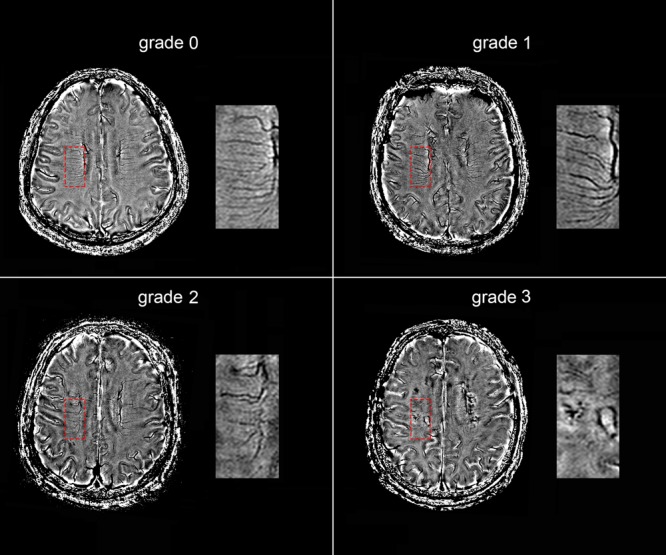
Deep medullary veins (DMVs) visual scores. An example of four-point DMVs score in parietal region: Grade 0 – each vein was continuous and had homogeneous signal; Grade 1 – each vein was continuous, but one or more than one vein had inhomogeneous signal; Grade 2 – one or more than one vein were not continuous, presented with spot-like hypointensity; Grade 3 – No observed vein was found continuous.

The following four-point score was used for the evaluation of DMVs: Grade 0 - each vein was continuous and had homogeneous signal; Grade 1 – each vein was continuous, but one or more than one vein had inhomogeneous signal; Grade 2 – one or more than one vein were not continuous, presented with spot-like hypointensity; Grade 3 – No observed vein was found continuous (**Figure 2**). Two neurologists (RZ and YZ, both with 3 years of neuroimaging review experience), who were completely blinded to the subjects’ clinical data and disease state, visually assessed the vascular changes.

### Volumetric Assessments of WMHs

The axial T2 FLAIR images were processed for the quantification of WMHs volume after it was coregistered to the phase images, according to the published methods (DeCarli et al., 1992, 1995). The segmentation threshold for WMHs was determined a priori as three standard deviations in pixel intensity above the mean of the fitted distribution of brain parenchyma as described previously (DeCarli et al., 1995; Wen and Sachdev, 2004). Then the automatically segmented lesions were manually checked and corrected by two experienced neuro-radiologists (XY and MZ) who were blinded to all other imaging and clinical data. The manual correction process included: (1) Correction of non-white matter area being labeled as WMH; (2) WMH area not properly labeled as WMH or normal appearing white matter falsely labeled as WMH. Since the intracranial volume (ICV) of each individual was different, we measured the corrected WMHs (cWMHs) volume for further analysis with the following equation: cWMHs volume = (WMHs volume × mean ICV)/ICV. The mean ICV is the mean value of ICV of all patients. The volume of periventricular hyperintensities (PVHs) and deep white matter hyperintensities (DWMHs) were measured, respectively (Fazekas et al., 1993; O’Sullivan et al., 2002), corrected by the ICV, too. PVHs volume was calculated 10 mm away from everywhere around the lateral ventricles for confluent type (Young et al., 2008). Additionally, the same six regions used for the above measurement of DMVs were used for the volumetric assessment of regional PVHs, which were defined as the sum of PVHs in each region, corrected by the ICV, too (**Figure 1**).

### Evaluation of Microbleeds and Lacunes

Microbleeds were identified according to a field guide of microbleeds detection and interpretation. SWI magnitude images were used to identify microbleeds. Briefly, microbleeds should be small, rounded or circular, well-defined hypointense lesions within brain parenchyma with clear margins ranging from 2 to 10 mm in size (Greenberg et al., 2009). Signal voids caused by sulcal vessels, calcifications, choroid plexus, and low-signal averaging from adjacent bone were excluded (Vernooij et al., 2008).

T2 FLAIR images were used to identify lacunes. Lacunes were defined as cavities with signal intensities similar to cerebrospinal fluid on MRI and with a diameter of 3 to 10 mm which was different from the enlarged Virchow–Robin spaces by the size, shape, and rim (Gouw et al., 2008).

### Reliability and Validity of the Radiological Measurements

All the raters were blinded to all other clinical and imaging data of the patients. A single-trained observer (RZ) performed the quantitative assessments of 50 patients twice, at an interval of 3 months apart. Another observer (YZ) independently made the measurements on the same patients. The interobserver intraclass correlation coefficients (ICCs) was 0.94 for grading of DMVs (including each region). The intraobserver ICCs was 0.91 for grading of DMVs (including each region). ICCs were described in detail elsewhere (Lee et al., 1989).

### Statistical Analysis

Patients were dichotomized according to WMHs volume or DMVs scores at the median. Comparison between groups were assessed by using Student’s *t*-test for data that followed normal distribution, Mann–Whitney *U* test for data that did not follow normal distribution, and Fisher’s Exact test for categorical data. We performed partial Pearson’s correlation analysis to determine the correlation between log-transformed corrected cWMHs volume, PVHs volume, DWMHs volume, and DMVs scores, by adjusting for age, and number of lacunes. We also conducted logistic regression analysis to provide an odds ratio statistic in order to facilitate comparison with other known risk factors. Backward stepwise conditional method was used to avoid the unstability resulted from collinearity of the included variables. The log-transformed cWMHs volume and DMVs scores appeared to be acceptably normative. All analyses were performed blinded to participant identifying information. A *p* value of <0.05 was considered to be statistically significant. All statistical analysis was performed with SPSS package (14.0 for Windows).

## Results

### Subject Characteristics

Totally, 268 consecutive WMHs patients were enrolled in this study. The main reasons for admission of those patients were transient ischemic attack (TIA) or lacunar ischemic stroke (*n* = 140, 52.2%), dizziness (*n* = 43, 16%), cognitive impairment (*n* = 18, 6.7%), gait disturbance (*n* = 9, 3.4%), anxiety or depression (*n* = 5, 1.9%), incidental finding of WMHs on MRI without symptoms (*n* = 53, 19.8%). Their age were 66.6 ± 11.5 years and cWMHs volume were 14.1 ± 16.3 mL. Among them, 160 (59.7%) subjects presented with microbleeds, and 110 (41.0%) subjects presented with lacunes.

### Comparison of the DMVs between WMHs and Controls

There were no significant differences in age and gender between WMHs patients and controls. The DMVs score in WMHs patients was significantly higher than controls [8 (6–11) vs. 0 (0–2), *p* < 0.001].

### Univariate and Multivariate Regression Analysis of the WMHs Volume

**Table 1** showed the baseline characteristics of patients for comparison. We classified the patients into mild cWMHs group (*n* = 134) and severe cWMHs group (*n* = 134) according to the cWMHs volume dichotomized at the median (8.36 mL).

**Table 1:** Basic data in different corrected WMHs (cWMHs) volume groups.

	cWMHs ≤ 8.36 mL (*n* = 134)	cWMHs > 8.36 mL (*n* = 134)	*p*-value
Age (Y)	66.26 ± 11.30	66.89 ± 11.76	0.657
Female (Y)	52 (38.8)	58 (43.2)	0.456
Years of education (Y)	6.04 ± 5.52	6.47 ± 5.29	0.690
MMSE	24.51 ± 6.21	23.81 ± 5.46	0.630
MoCA	21.42 ± 5.27	20.16 ± 6.88	0.406
Past medical history			
Hypertension	90 (67.2)	97 (72.4)	0.425
Diabetes mellitus	28 (20.9)	32 (23.9)	0.660
Hyperlipidemia	38 (28.4)	33 (24.6)	0.580
Clinical variables			
SBP (mmHg)	150.91 ± 23.01	148.53 ± 24.55	0.426
DBP (mmHg)	84.15 ± 13.52	83.75 ± 12.30	0.821
Glucose (mmol/L)	6.66 ± 2.84	5.72 ± 2.00	0.003
TC (mmol/L)	4.68 ± 0.99	4.12 ± 1.16	<0.001
THcy (μmol/L)	13.68 ± 8.30	15.08 ± 7.26	0.202
Hs-CRP (mg/L)	4.95 ± 8.33	7.64 ± 11.50	0.066
Radiology data			
No. of microbleeds	0 (0–1)	1 (0–5)	<0.001
No. of lacunes	0 (0–0.25)	1 (0–4)	<0.001
DMVs score	6 (6–8)	10 (8–14)	<0.001

*MMSE, mini-mental state examination; MoCA, Montreal cognitive scores; SBP, systolic blood pressure; DBP, diastolic blood pressure; TC, total cholesterol; THcy, total homocysteine; Hs-CRP, high-sensitive C-reactive protein; WMHs, white matter hyperintensities; cWMHs, Corrected WMHs; DMVs, deep medullary veins.*

Patients with severe cWMHs had higher DMVs scores (*p* < 0.001), more numbers of CMBs (*p* < 0.001) and more number of lacunes (*p* < 0.001), compared with those with mild cWMHs. Glucose and total cholesterol level were lower in patients with severe cWMHs.

Age, glucose level, total cholesterol level, DMVs score and number of lacunes were included in the primary logistic-regression model, and age, glucose level were then removed from the backward stepwise conditional model. The DMVs score was an independent factor for severe cWMHs (*OR* = 1.359; 95% CI: 1.224 to 1.508; *p* < 0.001), after adjusting for total cholesterol level and number of lacunes.

### Univariate and Multivariate Regression Analysis of the DMVs Score

We classified the patients into high DMVs scores group (*n* = 165) and low DMVs scores group (*n* = 103), according to the DMVs scores dichotomized at the median (8) (**Table 2**).

**Table 2:** Basic data in different DMVs score groups.

	DMVs score ≤ 8 (*n* = 165)	DMVs score > 8 (*n* = 103)	*p*-value
Age (Y)	65.95 ± 11.62	67.57 ± 11.34	0.269
Female (Y)	65 (39.4)	45 (43.7)	0.444
Years of education (Y)	6.50 ± 5.39	6.07 ± 5.39	0.685
MMSE	24.81 ± 5.75	23.34 ± 5.91	0.327
MoCA	22.23 ± 5.71	19.13 ± 6.21	0.040
Past medical history			
Hypertension	115 (69.7)	72 (69.9)	1.000
Diabetes mellitus	33 (20.0)	27 (26.2)	0.292
hyperlipidemia	47 (28.5)	24 (23.3)	0.395
Clinical variables			
SBP (mmHg)	149.40 ± 24.71	150.31 ± 22.22	0.769
DBP (mmHg)	83.94 ± 14.75	83.97 ± 12.36	0.989
Glucose (mmol/L)	6.64 ± 2.87	5.45 ± 1.45	<0.001
TC (mmol/L)	4.38 ± 1.13	4.37 ± 1.12	0.962
THcy (μmol/L)	14.55 ± 9.12	14.34 ± 5.74	0.846
Hs-CRP (mg/L)	4.68 ± 7.38	8.51 ± 12.59	0.008
Radiology data			
No. of microbleeds	0 (0–1)	1 (0–5)	0.001
No. of lacunes	0 (0–1)	1 (0–4)	<0.001
cWMHs volume (mL)	8.59 ± 10.80	23.04 ± 19.41	<0.001
PVHs volume (mL)	4.75 ± 4.90	11.63 ± 7.51	<0.001
DWMHs volume (mL)	3.83 ± 7.46	11.41 ± 13.94	<0.001

*PVHs, periventricular hyperintensities; DWMHs, deep white matter hyperintensities; other abbreviations same as **Table 2**.*

Patients with high DMVs scores had higher cWMHs volume, higher PVHs volume, and higher DWMHs volume, compared with those with low DMVs scores (all *p* < 0.001). Patients with high DMVs scores had more numbers of microbleeds (*p* = 0.018) and lacunes (*p* < 0.001). Glucose level were lower in patients with high DMVs scores (*p* < 0.001), and high-sensitivity C-reactive protein (hs-CRP) level were higher in patients with high DMVs scores (*p* = 0.008).

Since cWMHs volume, PVHs volume, and DWMHs volume were highly correlated with each other, they were included in the logistic-regression model separately. Finally, we found cWMHs volume (OR = 1.048, *p* = 0.001), PVHs volume (OR = 1.166, *p* < 0.001) and DWMHs volume (OR = 1.051, *p* = 0.007) were all independent factors for high DMVs scores, after adjusting for age, glucose level, Hs-CRP level and number of lacunes.

### Relationship between the DMVs and WMHs in Different Regions

Significant correlations were found between DMVs scores and cWMHs volume (Pearson *r* = 0.463, *p* < 0.001), PVHs volume (Pearson *r* = 0.539, *p* < 0.001) and DWMHs volume (Pearson *r* = 0.366, *p* < 0.001), respectively, after adjusting for age, glucose level, Hs-CRP level and number of lacunes.

**FIGURE 3:**
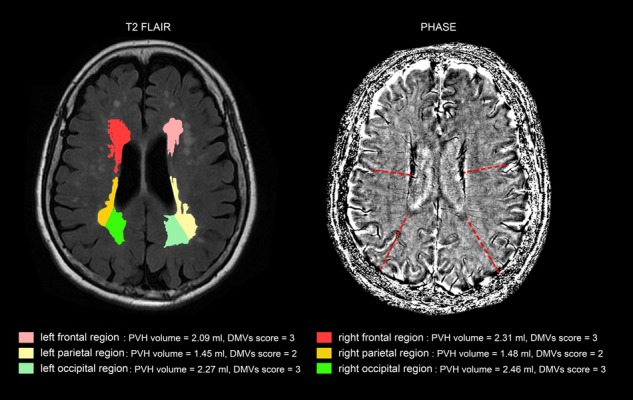
An example of correlation between deep medullary veins (DMVs) and periventricular hyperintensities (PVHs) in different brain regions. The PVHs in the subject in frontal regions and occipital regions were relatively severe than that of parietal regions, and the DMVs scores in frontal regions and occipital regions (both grade 3) were higher than that of parietal regions (grade 2).

Additionally, as **Table 3** shows, the correlation coefficient between DMVs scores and regional PVHs volumes in the same brain region was highest, except left frontal and right occipital region. **Figure 3** was an example figure illustrating the regional correlation between DMVs and PVHs.

**Table 3:** The correlation of DMVs and PVHs in different brain regions.

	Left frontal PVHs	Left parietal PVHs	Left occipital PVHs	Right frontal PVHs	Right parietal PVHs	Right occipital PVHs
Left frontal DMVs	**0.623**	0.545	0.450	0.631	0.554	0.302
Left parietal DMVs	0.359	**0.550**	0.314	0.379	0.543	0.349
Left occipital DMVs	0.438	0.475	**0.556**	0.454	0.480	0.414
Right frontal DMVs	0.601	0.516	0.448	**0.637**	0.527	0.329
Right parietal DMVs	0.366	0.542	0.304	0.378	**0.589**	0.375
Right occipital DMVs	0.402	0.447	0.547	0.420	0.440	**0.422**

*p-value < 0.05. PVHs, periventricular hyperintensities; DMVs, deep medullary veins. Bolded values are the Pearson r values between DMVs scores and PVHs volumes of the same brain regions.*

## Discussion

In the current study, we proposed a novel visual DMVs score based on brain lobes. We found an independent association between DMVs score and cWMHs volume, especially PVHs volume. Importantly, provided that DMVs mainly participate in the drainage of surrounding white matter, our finding that DMVs scores were correlated with regional PVHs volumes in the same brain region most may indicate that DMVs are involved in the pathogenesis of WMHs.

Postmortem MRI and histopathologic findings have revealed a noninflammatory, periventricular venulopathy with concentric collagen deposition in WMHs, which causes intramural thickening, stenosis and ultimately venous luminal occlusion (Moody et al., 1995; Black et al., 2009). Periventricular venous collagenosis was strongly associated with WMHs (Brown et al., 2002; Black et al., 2009). The visibility of the venous vessels on SWI depends on the de-oxygenation of the vessel (Rauscher et al., 2005), so the venous heterogeneous signal could be a result of altered venous hemodynamics or venous occlusion (Zeng et al., 2013). The venous outflow obstruction may contribute to the enlargement of WMHs due to ischemia. Furthermore, from the point of pathologic view, with the progress of intramural thickening and stenosis, luminal occlusion may become the end stage of DMVs disruption, contributing to the uncontinuous, spot-like hypointensity and decreased DMVs, and even no vein identified on SWI (Moody et al., 1995). It is thus rational to grade the DMVs based on both heterogeneous signal and the morphology of veins, but not purely on voxel analysis.

Our result supports the possible impact of DMVs drainage pattern on the surrounding white matter, which is consistent with a previous finding that there was relationship between the lesion location and the dilated veins in intracranial venous dysplasia patients, based on the anatomic analysis of the drainage of the medullary vein (Lee et al., 1996). In this study, we found that the correlation coefficient between DWMHs and DMVs scores was lower than that between PVHs and DMVs scores. This may be explained that part of DWMHs is drained by the superficial medullary veins. Moreover, the correlation coefficient of the same regional PVHs was higher than other regional PHVs, indicating that DMVs might have a direct influence on the surrounding white matter. Our brain region-based DMVs score may provide a tool for the future study to investigate the venous mechanism of WMHs.

We also found that patients with high DMVs scores had more lacunes. Lacunes most often result from arterial ischemia (Basile et al., 2006; Gouw et al., 2008), which may lead to high de-oxygenation of the vessel, to some extent. Previous studies demonstrated that WMHs are correlated with reduced cerebral metabolism (DeCarli et al., 1995) as well as reduced cerebral blood flow (CBF) on quantitative perfusion MRI (O’Sullivan et al., 2002). The reduced CBF would lead to the increased oxygen extraction from vessels and cause the subsequent venous heterogeneous signal on SWI, too (Yu et al., 2016). Moreover, venous outflow is influenced by the severity of reduction in arterial flow. It is thus reasonable to infer that the extent of upstream arterial ischemia also influences the change of DMVs. Further study to investigate the interaction between cerebral venous disruption and arterial perfusion may help to clarify it.

Our study had limitations. First, the signal to noise ratio (SNR) in SWI phase images is relatively lower than the SNR in minimum intensity projection (mIP) venograms. However, mIP images are lack of three-dimensional information of blood vessels. Besides, our assessment of DMVs was on a 3T brain MRI and the comparatively lower field strength may also reduce the quality of DMVs detection. Second, follow-up data was not available for most patients. Therefore, we cannot assess the natural progression of WMHs and the DMVs and not able to provide the causal relationship of DMVs and WMHs. Longitudinal and prospective studies are needed to clarify if the venous insufficiency contributes to the progression of white matter damage and whether DMVs disruption increases risks of stroke and cognitive decline. Third, although we used continuous thin-slice 3-T MRI scans and set the phase images as reference to minimize the effects of artifact, the step of coregistration may still bring artifact, especially for small structures like DMVs. Fourth, our cohort only included Chinese patients at a single institution who had distinct imaging changes or symptoms, and the exclusion criteria was strict. Therefore, it may not represent the full spectrum of WMHs, and the generalizability of our results need confirmation and extension in larger and multicenter cohorts.

In summary, our findings of cerebral venous insufficiency in WMHs patients could help to elucidate the pathogenic mechanisms and progression of WMHs. The newly proposed DMVs grading method may allow the clinician to monitor the course of DMVs disruption. Moreover, our results may provide insights in new therapies for WMHs targeting cerebral vein, such as to improve venous hemodynamics or increase the venous tone.

## Author Contributions

RZ and YZ drafted and revised the manuscript, participated in study concept and design, conducted the statistical analyses, analyzed, and interpreted the data. ML participated in study concept and design, data interpretation and made a major contribution in revising the manuscript. SY, GZ, and CL participated in the study design and made contribution in revising the manuscript. YJ, RS, XY, and MZ assisted in designing the MRI sequences and imaging analysis.

## Conflict of Interest Statement

The authors declare that the research was conducted in the absence of any commercial or financial relationships that could be construed as a potential conflict of interest.
